# Approach to An Unusual Cardiac Mass: Mitral Annulus Caseoma

**DOI:** 10.21470/1678-9741-2018-0361

**Published:** 2020

**Authors:** Arda Aybars Pala, Hasan Iner, Murat Abdulhamit Ercisli

**Affiliations:** 1 Cardiovascular Surgery, Adiyaman Universitesi Egitim ve Arastirma Hastanesi, Yunus Emre Mah., Adiyaman, Turkey.

**Keywords:** Mitral Valve, Mitral Valve Insufficiency, Debridement, Heart Valve Diseases, Cardiac Surgical Procedures

## Abstract

Caseous calcification of the mitral annulus (CCMA) is known to be a rare variant of mitral annulus calcification, a chronic and degenerative process of the mitral valve fibrous ring. It usually carries a benign prognosis. The following case demonstrates a huge mitral annulus caseoma that complicated with severe mitral regurgitation and was treated with a successful surgery. The common consensus on the optimal management of CCMA is conservative medical management and avoiding unnecessary surgery. Therewithal, the current indications for surgical intervention include mitral valve dysfunction, strokes and uncertain diagnosis. Aggressive debridement, risk of left ventricular perforation and exposure of caseous debris to the systemic blood flow may increase the risk of a standard mitral valve surgery. Mitral valve replacement should be preferred compared with mitral valve repair.

**Table:** 

Abbreviations, acronyms & symbols	
CCMA	= Caseous calcification of the mitral annulus
MVR	= Mitral valve replacement
TEE	= Transesophageal echocardiography
TTE	= Transthoracic echocardiography

## INTRODUCTION

Caseous calcification of the mitral annulus (CCMA) is known to be a rare variant of mitral annulus calcification, a chronic and degenerative process of the mitral valve fibrous ring^[1]^. It seems common in the elderly, particularly in women. It usually carries a benign prognosis and current data suggest conservative therapy and follow-up unless complicated with mitral valve dysfunction (mitral valve stenosis/mitral regurgitation) or systemic embolization. Also, it can resolve spontaneously, so the key point of managing this entity is avoiding unnecessary surgery. In our case, the indications to operate were severe mitral regurgitation despite optimal medical therapy and repeated pulmonary edema. With the patient's written informed consent, the following case demonstrates a huge mitral annulus caseoma complicated with severe mitral regurgitation and requiring surgery, presented to raise awareness for this entity and focus on the surgical indication and surgery techniques for CCMA.

## CASE REPORT

**Fig. 1:**
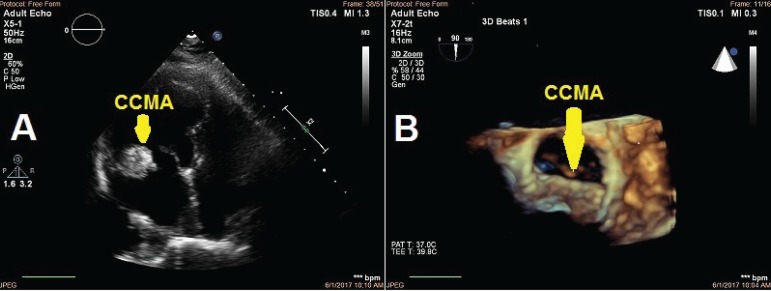
A) Two-dimensional transthoracic echocardiography (in the apical four-chamber view) of a calcified caseous mass attached to the mitral annulus (arrow). B) Three-dimensional transesophageal echocardiography of an irregular surface of CCMA (arrow).

**Fig. 2:**
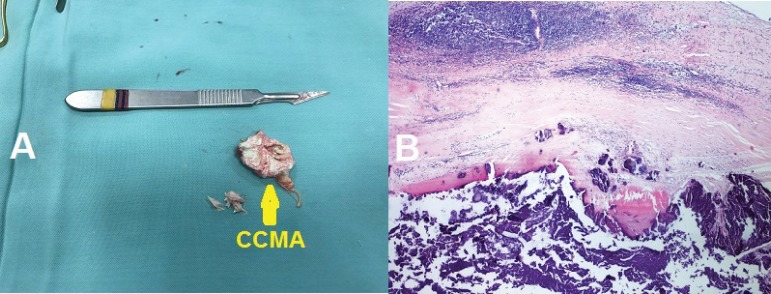
A) Postoperative photograph of the posterior mitral valve leaflet removed with CCMA (arrow). B) Microscopic examination of CCMA (hematoxylin and eosin, magnification of 100×).

An 87-year-old woman with a previous history of hypertension was referred for evaluation of a mass complicated with severe mitral regurgitation despite optimal medical therapy and possible surgery. Transthoracic echocardiography (TTE) and transesophageal echocardiography (TEE) showed a rounded mass of 29×27 mm in the posterior mitral annulus that extended to the most basal area, complicated with severe mitral regurgitation (Figure 1A). Also, the mass had a central area of echolucency resembling liquefaction and no flow was found in the central zone by color Doppler. The 3D TTE and 3D TEE examinations also confirmed that the mass appeared as a large area of liquefaction necrosis, with an irregular surface in the posterior mitral annulus (Figure 1B). The mass was diagnosed as caseous calcification of the mitral annulus with these morphological features and, surprisingly, CCMA had induced symptomatic severe mitral regurgitation in the patient. This case was discussed in the multidisciplinary meeting and decided for resection of the mass due to mitral valve dysfunction. A median sternotomy was performed. Then, after establishing cardiopulmonary bypass and cardioplegia, the superior septal approach to the mitral valve was performed and a white caseous material was exuded from the ventricular surface of the posterior mitral valve leaflet (Video clip). Due to the increased risk of left ventricular perforation, mitral valve replacement (MVR) was preferred to mitral valve repair and aggressive debridement was not performed. Surgical excision also provided a definitive diagnosis. Macroscopic view showed a large amount of putty-like material (Figure 2A) and microscopic examination revealed an amorphous eosinophilic acellular material surrounded by macrophages and lymphocytes (Figure 2B). The patient was discharged from the hospital on the 6^th^ day with good clinical status. During 6 months of follow-up, no recurrence of cardiac mass has been recognized.

**Video:**
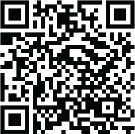
Intraoperative view of the removal of putty-like material from the ventricular surface of the posterior mitral valve leaflet.

## DISCUSSION

The prevalence of CCMA is about 0.06%-0.07% of the population^[1]^. CCMA is usually diagnosed incidentally and can be confused with other intracardiac masses such as cardiac tumors, abscesses, vegetation or calcified thrombi. Multimodality imaging, including TEE, cardiac computed tomography and cardiac magnetic nuclear resonance can easily differentiate CCMA from other masses and help avoiding unnecessary surgery. CCMA is typically located in the basal area of the posterior mitral valve and the calcification seems as a round, large, soft mass with a central echo-dense area. Although there is no consensus on the optimal management for CCMA, current data suggest conservative medical management for CCMA when there is no mitral valvular dysfunction (stenosis or regurgitation) or embolic manifestations. Valve dysfunction (valve stenosis/mitral regurgitation) or systemic embolization are an indication for curative surgery and for preventing recurrence of strokes. Although there are successful examples of both mitral valve repair and replacement, MVR is the most appropriate choice compared to mitral valve repair^[2,3]^. Davidson and Cohn cited an increased risk of left ventricular perforation with aggressive debridement. At the time of surgery, the mass may extend into the left atrial wall or into the ventricular endocardium. Thus, unroofing of the cavity may continue to expose necrotic debris to the systemic blood flow. MVR could provide support for this area and prevent ventricular rupture^[4]^.

## CONCLUSION

In conclusion, the common consensus on the optimal management for CCMA is conservative medical management and avoiding unnecessary surgery. Therewithal, current indications for surgical intervention include mitral valve dysfunction, stroke and uncertain diagnosis. Due to aggressive debridement, risk of left ventricular perforation and exposure of caseous debris to the systemic blood flow, it should be noted that the risk of a standard mitral operation is increased^[5]^.

**Table:** 

Authors’ roles & responsibilities	
AAP	Substantial contributions to the conception or design of the work; or the acquisition, analysis, or interpretation of data for the work; final approval of the version to be published
HI	Substantial contributions to the conception or design of the work; or the acquisition, analysis, or interpretation of data for the work; final approval of the version to be published
MAE	Final approval of the version to be published
